# Trajectories of daily antipsychotic use and weight gain in people hospitalized for the first episode of psychosis

**DOI:** 10.1192/j.eurpsy.2024.1761

**Published:** 2024-09-26

**Authors:** Kristyna Vochoskova, Sean R. McWhinney, Marketa Fialova, Marian Kolenic, Filip Spaniel, Petra Furstova, Petra Boron, Yurai Okaji, Pavel Trancik, Tomas Hajek

**Affiliations:** 1 National Institute of Mental Health, Klecany, Czech Republic; 2 Third Faculty of Medicine, Charles University, Prague, Czech Republic; 3 Department of Psychiatry, Dalhousie University, Halifax, NS, Canada; 4 Psychiatric Hospital Bohnice, Prague, Czech Republic

**Keywords:** antipsychotics, polypharmacy, predictor, schizophrenia, weight gain

## Abstract

**Background:**

We need to better understand the risk factors and predictors of medication-related weight gain to improve metabolic health of individuals with schizophrenia. This study explores how trajectories of antipsychotic medication (AP) use impact body weight early in the course of schizophrenia.

**Methods:**

We recruited 92 participants with first-episode psychosis (FEP, *n* = 92) during their first psychiatric hospitalization. We prospectively collected weight, body mass index (BMI), metabolic markers, and exact daily medication exposure during 6-week hospitalization. We quantified the trajectory of AP medication changes and AP polypharmacy using a novel approach based on meta-analytical ranking of medications and tested it as a predictor of weight gain together with traditional risk factors.

**Results:**

Most people started treatment with risperidone (*n* = 57), followed by olanzapine (*n* = 29). Then, 48% of individuals remained on their first prescribed medication, while 33% of people remained on monotherapy. Almost half of the individuals (39/92) experienced escalation of medications, mostly switch to AP polypharmacy (90%). Only baseline BMI was a predictor of BMI change. Individuals in the top tercile of weight gain, compared to those in the bottom tercile, showed lower follow-up symptoms, a trend for longer prehospitalization antipsychotic treatment, and greater exposure to metabolically problematic medications.

**Conclusions:**

Early in the course of illness, during inpatient treatment, baseline BMI is the strongest and earliest predictor of weight gain on APs and is a better predictor than type of medication, polypharmacy, or medication switches. Baseline BMI predicted weight change over a period of weeks, when other traditional predictors demonstrated a much smaller effect.

## Introduction

Obesity is more prevalent among people with schizophrenia than in the general population [1, 2] and contributes to the higher risk of cardiovascular mortality in this group [3]. While there are other causes, antipsychotic medications (AP) are a key contributor to weight gain and one which, unlike most other factors, is completely under the control of the psychiatrist [4, 5]. Therefore, we need to better understand the risk factors for medication-related weight gain.

Traditional risk factors for weight gain include type of medication, duration of treatment, dose, antipsychotic-naive status, lower age, female gender, nonsmoking status, high parental body mass index (BMI), and baseline BMI [6–12]. We know very little about other clinically highly relevant predictors, such as how medication switches and combinations of medications impact weight. This pertains to the contrast between the strict treatment protocols in randomized controlled trials (RCTs) and the realities of everyday clinical practice, where AP polypharmacy and switches of medications are common [13–15]. Protocols of RCTs usually emphasize one-to-one comparisons or focus on the transition from one monotherapy to another [16–19]. This does not tell us about the polypharmacological scenarios that are dominant in clinical practice. To learn about the impact of combining and switching medications on weight gain, we ultimately need data from everyday clinical practice, which are scarce.

Some real-life studies show limited impact of switches on weight gain [20, 21], while others suggest that both the switch itself and the AP previously administered may contribute to the metabolic outcomes observed in our patients [22–24]. These studies mostly focus on outpatients, where compliance is often an issue. They also frequently lack information on the duration of previous treatment, initial medication, and dosing [20–24]. Also a substantial portion of these studies investigated switches from medications with pronounced metabolic side effects to those with smaller impact on metabolism – for example, transitions from olanzapine and clozapine to ziprasidone or aripiprazole [20, 23, 25]. Such switches are rarely seen in clinical practice. In addition, we have very few studies which capture early stages of treatment, where the weight changes may be most pronounced [12, 26]. Only studies in people early in the course of treatment may speak to prevention and identify early predictors of medication-related weight gain.

Thus, we designed this study to fill the abovementioned knowledge gaps, specifically focusing on how sequences, combinations, and switches of medications impact weight gain early in the course of illness. To this goal, we prospectively collected daily medication records and measures of weight in people hospitalized for their first episode of psychotic illness. Inpatient setting allowed us to maximize adherence, precisely measure daily exposure to medication, and gain a snapshot of everyday inpatient psychiatric practice in a large general psychiatric hospital. Using these high-density data, we quantified the trajectory of medication changes taking the sequence, exact duration, dosing, overlaps of medications into account, and tested their effect on weight gain.

## Methods

### Materials and methods

Between 2016 and 2021, the Early-Stage Schizophrenia Outcome (ESO) study recruited participants from 10 psychiatric hospitals in the Czech Republic. Most of our patients (83%) came from one large psychiatric hospital with 1135 beds serving the Prague and Central Bohemian regions. We focused on individuals with first-episode psychosis (FEP), who met the following inclusion criteria: (a) were undergoing their first psychiatric hospitalization; (b) had the ICD-10 diagnosis of SZ (F20), or acute and transient psychotic disorders (F23) made by a psychiatrist according to Mini-International Neuropsychiatric Interview; (c) had <24 months of untreated psychosis; and (d) were 18–35 years old. We wanted to recruit participants at the early stages of illness to minimize the effects of illness and medications. Thus, participants who were hospitalized before meeting the duration criteria for schizophrenia were included in the study and received the working diagnosis of acute and transient psychotic disorders, which is congruent with the DSMIV brief psychotic disorder. All participants provided written informed consent for the study protocol approved by the Ethics Committee at the National Institute of Mental Health in Klecany. The treating psychiatrist evaluated the patients*’* capacity to provide informed consent before they were approached by the study team.

### Study procedures

Participants were assessed using the Positive and Negative Symptom Scale (PANSS). Weight and height were measured at the time of assessment by trained clinicians. BMI was calculated using the standard formula: BMI = weight (kg)/height (meters)^2^. Concurrent somatic illness as well as history of substance use, including smoking, alcohol, and other recreational drugs, were assessed in a structured interview with the study psychiatrist. Subsequently, using the inpatient charts, we collected data from the time of admission, including BMI, and calculated the duration of inpatient treatment, that is, the interval between admission and research visit in days. Using the daily chart notes, we recorded daily medication exposure, calculated the precise doses for every administered antipsychotics, and ranked AP medications based on the strength of their association with body weight using whole numbers from 1 to 12 according to recent network meta-analyses [11, 27, 28] (see Supplementary Material for details).

### Quantifying polypharmacy

**Figure 1.**
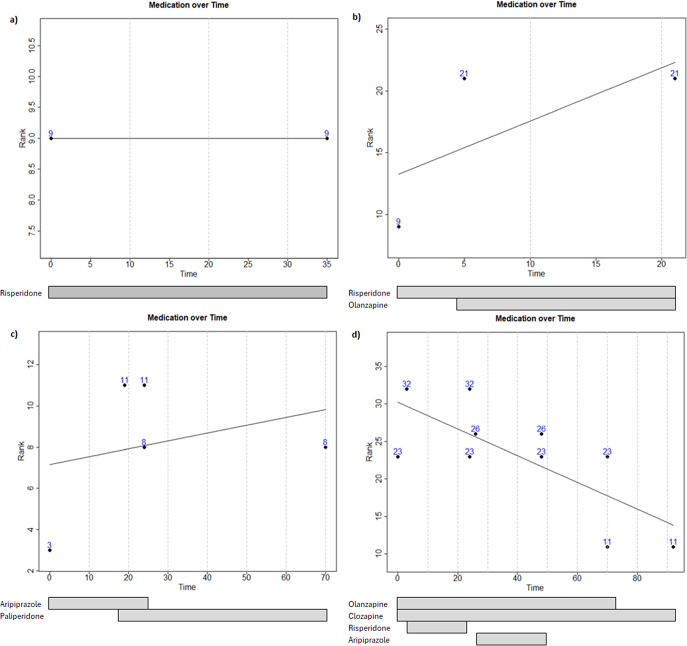
Examples of polypharmacy plots for four participants: (A) shows a 35 day follow-up of an individual with FEP on risperidone monotherapy (rank 9); (B) shows a 20 day follow-up of an individual with FEP on risperidone during the initial 5 days of hospitalization, olanzapine (rank 12) was added on the 5th day; (C) shows a 70 day follow-up of an individual with FEP initially prescribed aripiprazole (rank 3) from days 1 to 24, on day 19, paliperidone (rank 8) was added until day 70, the cumulative rank (11) is the sum of ranks 3 and 8; (D) 92 day follow-up of an individual with FEP, olanzapine was administered between days 1 and 70, clozapine between days 1 and 92, risperidone between days 3 and 24, and aripiprazole between days 26 and 48.

We calculated the trajectory of AP polypharmacy by plotting the rank of medication (*y*-axis) over time in days (*x*-axis) with points at the time of prescription and termination of each medication (see Figure 1). The rank of subsequent medications in plotting was summed with the rank of any medications that were active at the time of their prescription or discontinuation. Termination of medications was noted with an additional point at the summed rank of all remaining medications. A regression line was fitted through these points, and the intercept and slope were extracted as a measure of the metabolic impact of the initial prescription, and the change in metabolic impact throughout treatment, respectively. In order to separately account for the dose of medications, we repeated the above-described procedure using the average standardized daily dose, instead of rank, for each medication. Even if medications were used short time or as needed, we accounted for them when quantifying the medication-related slopes and intercepts. The result was an intercept and slope for each medication rank and dose. These four measures were used as predictors of change in weight or BMI, described below.

We also performed alternative analyses, where we included average weight gain per medication as estimated in meta-analyses [11, 27, 28]. However, this was not consistently done for the first generation of antipsychotics – consequently, we assigned the same number to all these medications. Also, the duration of studies used to calculate this markedly differed. We then recalculated polypharmacy trajectories using average weight gain associated with each medication instead of medication ranking. As this analysis is based on more assumptions and more heterogeneous set of studies, we only include this information in the supplement, as a sensitivity check (see Supplementary Table S1). Among the individuals who were on more than one medication during the hospitalization, 93.5% were on polypharmacy. Consequently, we could not compare metabolic switches versus polypharmacy.

### Statistical analyses

We used linear multiple regression modeling to test whether baseline BMI or change in BMI over time, were associated with a number of predictors. Baseline BMI was tested for association with the intercept of medication rank and dose. Change in BMI was tested for associations with the slope and the intercept of both the medication rank and dose, as well as baseline BMI, and treatment duration. We controlled for age and sex in all of the models. We used linear regression modeling to test for associations between changes in BMI with PANSS scores (positive, negative, and global) while controlling for age, sex, duration of treatment, and medication dose intercept.

Residuals were confirmed as normally distributed using the Kolmogorov–Smirnov test for normality, as well as visually using QQ plots. Multicollinearity was tested by calculating the variance inflation factor among all predictors in a model and was negligible.

Finally, participants were split into those whose change in BMI was in the top or bottom 33%, and the two groups were compared in demographic and clinical variables using either pairwise two-tailed *t* tests for continuous variables or Chi-square testing for categorical variables. This same procedure was completed to compare participants with a positive medication rank slope relative to those with a negative slope. In addition, to check for regression to the mean, we performed a median split based on the baseline BMI and compared the BMI change in each subgroup.

We performed a retrospective power analysis for each effect shown in Table 2, using an effect size as calculated from the *t*-statistic for the respective effects, a confidence level of 0.95, and a power level of 0.80. In each case, we determined the number of observations required to achieve this power and confidence level given the present effect size.

## Results

### Sample

The sample included 92 participants (70.7% male) with a mean age of 25.2 years (SD = 4.9). More than half (59.8%) of the participants were medication naïve, and the onset of symptoms was on average 4.58 months prior to the study date (SD = 4.60). In participants with prior medication, the average duration of prehospitalization treatment with antipsychotics was 4.27 months (SD = 6.12). Participants had a mean baseline BMI at hospitalization of 22.84 (SD = 4.40), and a mean BMI at follow-up of 23.70 (SD = 4.25). Those with BMI recorded at both times experienced a statistically significant average increase of 0.74 (SD = 1.84) between admission and the research visit (*t*(77) = 3.56, *p* < 0.001) and we observed clinically significant weight gain (≥7%) in 30.4% of participants. Within our sample, none of the participants had a personal history of type 2 diabetes mellitus or hypertension; one participant was diagnosed with and treated for hypothyroidism, two were chronically treated for allergies, and one was treated for gastroesophageal reflux disease. Additionally, none of the participants exhibited signs of hypertension or diabetes in their blood work and blood pressure measurements. Among the participants, 51.2% reported nicotine use, and none had a prior diagnosis or treatment for substance use disorder at the time of admission.

### Prescription patterns

The numbers of people prescribed each of the following medications as their first medication after admission were: risperidone (*n* = 57), olanzapine (*n* = 29), quetiapine (*n* = 3), aripiprazole (*n* = 2), and clozapine (*n* = 1). The total number of different medications used was 15 (mea*n* = 2.52 medications per individual, SD = 1.68) in our sample. More than half of the individuals (57%) who received risperidone or olanzapine were additionally treated with another AP medication during hospitalization. Less than half of the individuals (44/92) remained on their first prescribed medication for the whole duration of follow-up (for 33.1 days [SD = 17.1], with a median of 31 days). The average duration before the first change of medications (switch or addition of a new medication), was 17.6 (SD = 17.9, median =10) days.

**Figure 2.**
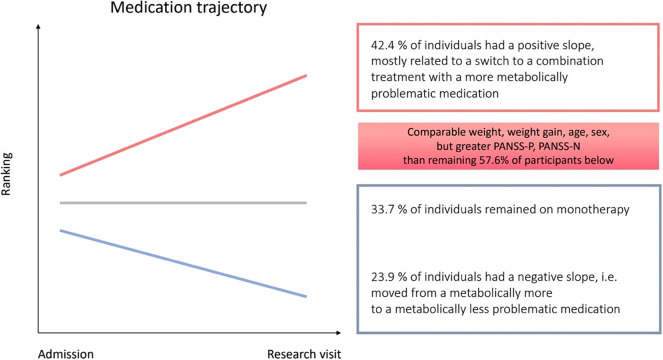
Comparison of AP medication trajectories from admission to the research visit and their characteristics.

One third of individuals (32.6%) remained on a monotherapy for the entire duration of follow-up (33.1 [SD = 17.1] days, median 31 days). Almost half of the individuals (42.4%) had a positive slope for ranking of meds based on metabolic side effects. This was related to switch to a combination treatment: N = 12, switch to a metabolically more problematic medication: N = 4, and a switch to a combination treatment with a more metabolically problematic medication: N = 23. Therefore, 35/39 individuals in this group (~90%) moved to treatment with polypharmacy. Only 23.9% of individuals (*n* = 22) had a negative slope, that is, moved from a metabolically more to a metabolically less problematic medication. Relative to those with negative slope, individuals with positive slope had greater PANSS scores for positive and negative symptoms at follow-up (see Table 1), indicating possibly that people with more symptoms/worse response needed escalation of medication (see Figure 2). Twenty-six of 92 participants (28.2%) were treated with antidepressants, 5 individuals took valproic acid, 1 lithium, and 1 modafinil.

**Table 1.** Demographic and clinical characteristics of participants with either a positive or a negative medication rank slope (*, *p* < 0.05)

	Medication rank slope		
	Negative or zero	Positive	Significance
Sample size	53	39	
Age – Mean (SD)	25.84 (5.02)	24.42 (4.71)	*t*(90) = −1.38, *p* = 0.172
Sex, male – No. (%)	37 (69.8)	28 (71.8)	χ^2^ = 0.01, *p* = 1.000
Drug–naive – No. (%)	32 (60.4)	23 (59.0)	χ^2^ = 0.01, *p* = 1.000
Baseline BMI – Mean (SD)	23.48 (4.88)	22.00 (3.58)	*t*(77) = −1.49, *p* = 0.139
Follow–up BMI – Mean (SD)	24.23 (4.84)	22.98 (3.16)	*t*(89) = −1.39, *p* = 0.167
Change in BMI – Mean (SD)	0.67 (1.97)	0.83 (1.67)	*t*(76) = 0.37, *p* = 0.709
PANSS Global – Mean(SD)	28.48 (7.38)	31.24 (8.29)	*t*(88) = 1.66, *p* = 0.100
PANSS Positive – Mean (SD)	10.25 (3.33)	12.03 (3.24)	*t*(88) = 2.53, *p* = 0.013*
PANSS Negative – Mean (SD)	15.50 (5.40)	18.71 (6.04)	*t*(88) = 2.65, *p* = 0.010*
Weighted medication rank sum – Mean (SD)	434.17(326.19)	668.21 (457.37)	*t*(90) = 2.87, *p* = 0.005*
Weighted medication gain sum – Mean (SD)	108.73 (96.20)	186.02 (137.34)	*t*(90) = 3.18, *p* = 0.002*
Proportion AP problematic – Mean (SD)	0.70 (0.33)	0.60 (0.22)	*t*(66) = −1.40, *p* = 0.167
Treatment prior to hospitalization – Mean (SD)	2.10 (4.13)	1.48 (0.87)	*t*(88) = −0.90, *p* = 0.368

### Impact of prescription patterns on baseline weight or weight gain

Baseline BMI was not associated with the slope or intercept of medication ranking or dose (see Table 2) or with the initial dose (*t*(74) = −1.21, *p* = 0.231) or the initial medication’s rank (*t*(74) = −0.13, *p* = 0.897), suggesting that weight was not a factor in selecting the initial medication. When we jointly analyzed the main predictors of weight gain, age, sex, duration of treatment, and baseline BMI with the new measures, including slope and intercept for dose, rank, only low baseline BMI was a predictor of greater BMI increase (see Table 2). Sex did not influence the effects of rank slope (F(1,65) = 2.39, *p* = 0.127), rank intercept (F(1,65) = 0.01, *p* = 0.939), dose slope (F(1,65) = 0.49, *p* = 0.485), or dose intercept (F(1,65) = 053, *p* = 0.471), on weight gain. Taking a median split on baseline BMI, the lower 50% of participants gained on average 1.53 points (SD = 2.28). The higher 50% of participants also gained weight, on average gaining 0.40 points (SD = 2.12). Notably, the slope or intercept of medication ranking, or dose were not significantly associated with change in BMI (see Table 2). Retrospective power analysis shows that effects of medication rank would not be significantly associated with change in BMI, even at a much higher sample size, that is, thousands of participants, thus suggesting this to be a true negative finding (see Table 2).

**Table 2.** Predictors of baseline BMI, change in BMI, and change in weight (*, *p* < 0.05)

Outcome	Predictor	Estimate (SE)	Standardized coefficient (SE)	Significance	Sample required for power 0.80
Baseline BMI	*Rank intercept*	0.080 (0.142)	*0.068 (0.119)*	*F*(1,74) = 0.32, *p* = 0.573	1838
	*Dose intercept*	0.001 (0.001)	*−0.211 (0.118)*	*F*(1,74) = 3.18, *p* = 0.079	187
	*Age*	0.232 (0.103)	*0.246 (0.109)*	*F*(1,74) = 5.11, *p* = 0.027*	117
	*Sex (M)*	2.961 (1.046)	*0.340 (0.120)*	*F*(1,74) = 8.01, *p* = 0.006*	75
BMI change	*Medication rank slope*	−0.469 (1.569)	*−0.079 (0.263)*	*F*(1,69) = 0.09, *p* = 0.766	6052
	*Medication rank intercept*	−0.029 (0.065)	*−0.105 (0.237)*	*F*(1,69) = 0.02, *p* = 0.658	2748
	*Dose slope*	−0.001 (0.001)	*−0.150 (0.219)*	*F*(1,69) = 0.47, *p* = 0.495	1151
	*Dose intercept*	0.001 (0.001)	*0.280 (0.307)*	*F*(1,69) = 0.83, *p* = 0.364	651
	*Baseline BMI*	−0.115 (0.052)	*−0.497 (0.225)*	*F*(1,69) = 4.89, *p* = 0.030*	113
	*Treatment duration*	0.007 (0.013)	*0.164 (0.297)*	*F*(1,69) = 0.31, *p* = 0.582	1770
	*Age*	−0.027 (0.047)	*−0.123 (0.218)*	*F*(1,69) = 0.32, *p* = 0.574	1697
	*Sex (M)*	0.623 (0.488)	*0.310 (0.243)*	*F*(1,69) = 1.63, *p* = 0.206	334

*Note*: The sample size required to achieve a power of 0.80, given the size of each effect, is shown.

There was a positive association between dose intercept versus rank intercept (*t*(90) = 3.18, *p* = 0.002). That is, when treating clinicians used a high dose, they also used a more metabolically problematic medication.

Medication-naive participants gained on average 0.98 BMI points (SD = 2.27), while those with treatment history gained 0.94 (SD = 2.27). Being medication-naive had no significant influence on weight gain when controlling for baseline BMI, age, sex, and duration of treatment (F(1,72) = 0.21, *p* = 0.652). Being prescribed an antidepressant was not significantly associated with change in BMI (*t*(75) = 0.78)), *p* = 0.438). The use of other medications was so low to prevent any systematic analyses of their associations with weight gain.

### Comparison of extremes

Relative to the third of individuals who gained the least amount of weight (mean BMI change of –1.02, SD = 0.87), the third of individuals who gained the most weight (mean BMI change of 3.24, SD = 2.25) had significantly lower global, positive and negative symptom scores at the end of the follow-up, a trend for longer prehospitalization antipsychotic treatment and a trend for greater exposure to medications with the highest potential for weight gain (expressed as weighted medication gain sum; see Table 3). However, when constrained to the same medications, change in BMI was not significantly associated with positive (*t*(66) = 1.28, *p* = 0.205); negative (*t*(66) = 1.80, *p* = 0.077); or global (*t*(66) = 0.04, *p* = 0.968) PANSS scores.

**Table 3.** Demographic and clinical characteristics of participants whose change in BMI during treatment was among the lowest and highest 33% (*, *p* < 0.05)

	Change in BMI		
	Bottom 33%	Top 33%	Significance
Sample size	26	28	
Age – Mean (SD)	25.10 (4.72)	23.66 (4.09)	*t*(52) = –1.19, *p* = 0.238
Sex, male – No. (%)	17 (65.4)	24 (85.7)	χ2 = 2.04, *p* = 0.153
Drug–naive – No. (%)	15 (57.7)	18 (64.3)	χ2 = 0.05, *p* = 0.828
Baseline BMI – Mean (SD)	23.86 (4.14)	22.01 (4.65)	*t*(52) = –1.54, *p* = 0.130
Follow–up BMI – Mean (SD)	22.84 (3.82)	25.25 (4.78)	*t*(52) = 2.04, *p* = 0.047*
Change in BMI – Mean (SD)	–1.02 (0.87)	3.24 (2.25)	*t*(52) = 9.03, *p*<0.001*
PANSS Global – Mean (SD)	33.40 (6.92)	26.50 (7.09)	*t*(51) = –3.58, *p* = 0.001*
PANSS Positive – Mean (SD)	12.40 (4.13)	10.04 (3.11)	*t*(51) = –2.37, *p* = 0.022*
PANSS Negative – Mean (SD)	18.08 (5.26)	14.79 (4.63)	*t*(51) = –2.43, *p* = 0.019*
Problematic AP, average daily dose – Mean (SD)	400 (218)	360 (221)	*t*(40) = −0.58, *p* = 0.567
Nonproblematic AP, average daily dose – Mean (SD)	465 (503)	308 (284)	*t*(40) = –1.27, *p* = 0.211
Problematic AP, cumulative exposure – Mean (SD)	12821 (9615)	16134 (13034)	*t*(40) = 0.92, *p* = 0.363
Nonproblematic AP, cumulative exposure – Mean (SD)	11381 (11514)	9322 (7751)	*t*(40) = −0.69, *p* = 0.494
Medication rank intercept – Mean (SD)	13.49 (3.98)	14.65 (4.10)	*t*(48) = 1.02, *p* = 0.315
Medication rank slope – Mean (SD)	0.04 (0.16)	0.04 (0.18)	*t*(48) = −0.04, *p* = 0.969
Medication dose intercept – Mean (SD)	13142 (8306)	16515 (13022)	*t*(48) = 1.09, *p* = 0.280
Medication dose slope – Mean (SD)	122 (191)	70 (222)	*t*(48) = −0.89, *p* = 0.380
Weighted medication rank sum – Mean (SD)	480.84 (260.64)	603.44 (440.11)	*t*(48) = 1.20, *p* = 0.237
Weighted medication gain sum – Mean (SD)	118.12 (65.32)	170.22 (137.43)	*t*(48) = 1.71, *p* = 0.093
Proportion AP problematic – Mean (SD)	0.60 (0.29)	0.62 (0.30)	*t*(40) = 0.21, *p* = 0.834
Treatment prior to hospitalization – Mean (SD)	1.39 (1.73)	3.36 (4.93)	*t*(51) = 1.92, *p* = 0.060

## Discussion

This study provides ecological insights into inpatient treatment of FEP and its impact on weight. Most individuals admitted to the hospital for the first episode of psychosis started treatment with either risperidone or olanzapine. Less than half of the individuals remained on the same medication throughout their first hospitalization, and only a third of people with FEP remained on monotherapy. The first switch or addition of new medication happened within a median of 10 days. Almost half of the individuals (42.4%) experienced an escalation of medications, mostly a switch to a medication with a worse metabolic profile than their initial treatment. De-escalation of medications, that is, switch to a medication with less propensity for metabolic changes was rare, happened only in 23.9% of individuals. The medication choices and changes appeared to be primarily related to symptoms and were unrelated to baseline BMI. Among the potential predictors, only lower baseline BMI was associated with greater weight gain. Slope or intercept for medication rank or dose, which reflect the patterns of medication changes were not related to weight gain and would not be even with thousands of individuals. Even when we expressed the metabolic burden of medications as average weight gain reported in previous meta-analyses, the results remained the same.

The observation that baseline BMI might serve as a predictor for subsequent weight gain during antipsychotic treatment is an extensively replicated finding. Baseline BMI is a predictor of weight gain over long (3–5 years [8, 9]) intermediate (39–52 weeks, [7, 10]) or even relatively short term of 3 months [29, 30] in people with schizophrenia spectrum disorders. Importantly, our study is the first to demonstrate that baseline BMI predicts weight gain in naturalistic setting, over a very short period of 6.3 weeks in a population experiencing their first episode of psychosis. This did not appear to be a simple regression to the mean, as individuals in both the higher and lower median gained weight.

Our study provides support for two other predictors of weight gain, type of medication, and duration of medication treatment. After on average weeks of treatment, the effect of these factors was evident, but only as trend and when comparing extremes of weight change. People who gained the most weight had a trend for greater exposure to metabolically more problematic meds or a longer duration of prehospitalization treatment than people who lost weight. This is in line with many previous studies [4, 27, 31, 32]. It is likely that over time the effect of the type of medication and duration of treatment would become more pronounced and would not be evident only when comparing extremes of weight change. This further suggests that baseline BMI is a faster and more general predictor than type of medication or duration of treatment. In addition, there is little we can do about the duration of treatment or the selection of medication, which are mostly dictated by the severity/type of illness. However, we can screen for people with baseline low BMI and be particularly cautious in treating those, by prescribing them medications with the lowest potential for weight gain.

In our study, the starting point or trajectory of medication changes was not related to weight gain after an average of 6.3 weeks of treatment. This is in contrast to studies, where transitioning to olanzapine for 3 months to 1 year was associated with significant weight gain [33, 34]. In a before-to-after switch meta-analysis, there was no statistically significant change evident when switching to amisulpride, quetiapine, paliperidone, risperidone, or lurasidone [17]. This is similar to our findings. In contrast, a significant increase in weight was noted when switching to olanzapine (+2.7 kg) and clozapine (+2.8 kg) [17], but the mean study duration was 26.3 weeks compared to 6.3 weeks in our study. With these longer intervals, we are essentially seeing the effect of the prolonged use of the new medication. This is in keeping with our findings, where individuals who experienced the most pronounced weight gain had greater exposure to metabolically more problematic meds than people with the lowest weight gain. We can conclude from this that the switch itself even to a more metabolically problematic medication is not causing weight gain over a relatively short period of time (weeks to months). In the short term, baseline BMI was a more sensitive predictor of weight gain than medication changes.

We observed an association between weight change and symptoms, such that individuals who gained the most weight showed fewer symptoms at follow-up than individuals who lost weight. This association was documented in other studies [35–38]. However, when constrained to the same medications, BMI change was not associated with any symptoms in any model. We only saw this effect in a strict comparison of the top and bottom 33% (i.e., Table 3) based on weight gain. However, people in these two groups were not treated with the same medications. So in our study, treatment response to a medication was not associated with weight gain. What we effectively observed was that individuals who gained weight had a trend for greater exposure to metabolically more problematic medications and fewer symptoms at follow-up; that is, a more aggressive treatment led to both a greater weight gain and fewer symptoms. This seems in keeping with the presumably greater efficacy of metabolically more (-ines) versus metabolically less problematic (-oles) medications [27].

Our study provides an interesting snapshot of how closely the treatment in a large psychiatric hospital conforms with guidelines. In certain instances, the observed patterns were in line with guidelines. The majority of individuals with FEP in our study initiated treatment with risperidone (*n* = 57), followed by olanzapine (*n* = 29), which in in keeping with the Czech guidelines [39]. Other international guidelines, such as National Institute for Health and Care Excellence [40], Guidelines for the Pharmacotherapy of Schizophrenia in Adults, Canada [41], and APA guidelines [42] do not recommend specific medications in individuals with FEP, but only advocate for consideration of side effects. In contrast, Royal Australian and New Zealand College of Psychiatrists clinical practice guidelines for early psychosis [43] and Swiss guidelines for early psychosis [44] treatment caution against olanzapine as the first-choice medication, recommending it only as a second-line option. Guidelines, to some extent, do seem to determine treatment in a given country. It is thus concerning that there are such vast differences between guidelines when all should be based on the same evidence. Greater attempts should be made to synchronize the guidelines and make them more closely reflect the most up to date evidence.

Our study also reveals a notable discrepancy between clinical practices and guideline recommendations. Approximately two-thirds of individuals in our sample were treated with AP polypharmacy. This is in keeping with some other studies from Europe, Canada, and the United States, which have reported polypharmacy rates ranging widely from 19% to 67% [13, 45–49]. While there is evidence supporting the potential superiority of antipsychotic cotreatment over monotherapy in certain clinical situations, such as combining aripiprazole with clozapine associated with the lowest risk of rehospitalization [49, 50], the current recommendations for first-line treatment in early psychosis continue to emphasize antipsychotic monotherapy. Despite limited research on polypharmacy in FEP and the consequent absence of guideline recommendations, they remain the most frequent pattern in clinical practice.

This study has important clinical implications. Despite initiating treatment with metabolically problematic medications, a substantial number of individuals (40/92) experienced an escalation of medications, in 90% of cases, an addition of another medication. The main concern with using metabolically less problematic medications may be their presumably lower efficacy [51]. This is not necessarily supported by meta-analytical evidence [52, 53]. If these medications were less effective, we could expect even more switches if commencing treatment with metabolically less problematic options. However, it might not be a significant concern since early switches did not impact weight gain. Starting individuals on metabolically less problematic medications could offer a greater number of people a better chance of avoiding weight gain. For this reason, advocating for the less metabolically problematic medications as a first-line treatment early in the course of illness is warranted, even if they were less effective, which is not clear. Unfortunately, in some countries, including Czechia, insurance companies do not cover prescription of the safer medications to those with low to normal baseline BMI.

The strengths of this study include the real-life setting of a large psychiatric hospital, a range of medications, and a broad representation of individuals with psychotic disorders in novel prospective design. The inpatient setting minimizes possibility of medication nonadherence and dietary differences among individuals, thereby ensuring abstinence from other psychoactive substances. Research on early psychosis brings advantages and minimizes the impact of past medication exposure and illness, creating more sensitive circumstances for detection of medication-related alterations. In our study, all diagnostic and clinical assessments were conducted by clinical psychiatrists, thereby minimizing other potential sources of error. Furthermore, we had access to daily chart notes and had the opportunity to precisely measure medication exposure, which is uncommon in real-world studies on FEP. We applied a novel method, which allowed us to track the trajectory of prospectively tracked medication changes and evaluate their impact on weight.

This study has the following limitations. This was not an RCT. However, clinical practice may significantly differ from controlled trials, and certain questions may only be addressed in naturalistic designs. We can only assume the reasons for changes in medication, and there is a potential for patient/prescriber bias. Although our study provides a detailed and precise description of AP usage day by day, physical measures and psychopathology were assessed only at the end of the study during the research visit, thus limiting our temporal resolution. Physical activity, diet, substance abuse, and socioeconomic factors prior to hospitalization could not be controlled. We screened for illegal substance use and misuse, as well as alcohol and nicotine consumption, during a clinical assessment, but not by a standardized questionnaire. At the same time, information about substance abuses, especially in people who are acutely psychotic may not be accurate by any means. Of note, in the Czech Republic, smoking is allowed on psychiatric wards, so there was no change in smoking habits during hospitalization that could possibly lead to additional weight changes. Weight gain during hospitalization may be higher than it would be in an outpatient setting. The average duration of follow-up was relatively short, but it was sufficient for people to gain weight. In addition, it allowed us to establish the most sensitive, fastest predictor of weight changes.

To conclude, we demonstrated that early in the course of illness, during inpatient treatment, baseline BMI remains the strongest predictor of weight gain on APs and is a better predictor than type of medication, initial medication, polypharmacy, or medication switches. Remarkably, baseline BMI may also be one of the fastest predictors, as it predicted weight change over a very short period of time, that is, just weeks, when other traditional predictors did not yet demonstrate an effect. Type of medication or treatment duration early in the course of treatment are relevant only when comparing extremes of weight change, so are likely slower to impact weight gain than the baseline BMI. The study supports the use of metabolically less problematic medications as a first-line treatment early in the course of illness, especially in people with low baseline weight. This approach would give more people a greater chance to prevent/delay medication-related weight gain.

## Supporting information

Vochoskova et al. supplementary material Vochoskova et al. supplementary material

## Supplementary material

The supplementary material for this article can be found at http://doi.org/10.1192/j.eurpsy.2024.1761.

## References

[1] Afzal M, Siddiqi N, Ahmad B, Afsheen N, Aslam F, Ali A, et al. Prevalence of overweight and obesity in people with severe mental illness: systematic review and meta-analysis. Front Endocrinol. 2021;12:769309. doi:10.3389/fendo.2021.769309. PMC8656226 34899604

[2] Annamalai A, Kosir U, Tek C. Prevalence of obesity and diabetes in patients with schizophrenia. World J Diabetes. 2017;8:390. doi:10.4239/wjd.v8.i8.390. 28861176 PMC5561038

[3] Correll CU, Solmi M, Croatto G, Schneider LK, Rohani-Montez SC, Fairley L, et al. Mortality in people with schizophrenia: a systematic review and meta-analysis of relative risk and aggravating or attenuating factors. World Psychiatry. 2022;21:248–71. doi:10.1002/wps.20994. 35524619 PMC9077617

[4] Bak M, Fransen A, Janssen J, van Os J, Drukker M. Almost all antipsychotics result in weight gain: a meta-analysis. PLoS One. 2014;9:e94112. doi:10.1371/journal.pone.0094112. 24763306 PMC3998960

[5] Manu P, Dima L, Shulman M, Vancampfort D, De Hert M, Correll CU. Weight gain and obesity in schizophrenia: epidemiology, pathobiology, and management. Acta Psychiatr Scand. 2015;132:97–108. doi:10.1111/acps.12445. 26016380

[6] Bak M, Drukker M, Cortenraad S, Vandenberk E, Guloksuz S. Antipsychotics result in more weight gain in antipsychotic naive patients than in patients after antipsychotic switch and weight gain is irrespective of psychiatric diagnosis: a meta-analysis. PLoS One. 2021;16:e0244944. doi:10.1371/journal.pone.0244944. 33596211 PMC7888647

[7] Brecher M, Leong RW, Stening G, Osterling-Koskinen L, Jones AM. Quetiapine and long-term weight change: a comprehensive data review of patients with schizophrenia. J Clin Psychiatry. 2007;68:597–603. doi:10.4088/JCP.v68n0416. 17474816

[8] Bushe CJ, Slooff CJ, Haddad PM, Karagianis JL. Weight change by baseline BMI from three-year observational data: findings from the worldwide schizophrenia outpatient health outcomes database. J Psychopharmacol (Oxf). 2013;27:358–65. doi:10.1177/0269881112473789. 23343595

[9] Gebhardt S, Haberhausen M, Heinzel-Gutenbrunner M, Gebhardt N, Remschmidt H, Krieg J-C, et al. Antipsychotic-induced body weight gain: predictors and a systematic categorization of the long-term weight course. J Psychiatr Res. 2009;43:620–6. doi:10.1016/j.jpsychires.2008.11.001. 19110264

[10] Kinon BJ, Basson BR, Gilmore JA, Tollefson GD. Long-term olanzapine treatment: weight change and weight-related health factors in schizophrenia. J Clin Psychiatry. 2001;62:92–100. 11247108

[11] Pillinger T, McCutcheon RA, Vano L, Mizuno Y, Arumuham A, Hindley G, et al. Comparative effects of 18 antipsychotics on metabolic function in patients with schizophrenia, predictors of metabolic dysregulation, and association with psychopathology: a systematic review and network meta-analysis. Lancet Psychiatry. 2020;7:64–77. doi:10.1016/S2215-0366(19)30416-X. 31860457 PMC7029416

[12] Vochoskova K, McWhinney SR, Fialova M, Kolenic M, Spaniel M, Svancer P, et al. Weight and metabolic changes in early psychosis―association with daily quantification of medication exposure during the first hospitalization. Acta Psychiatr Scand. 2023;148:265–76. doi:10.1111/acps.13594. 37528692

[13] Farrell C, Brink J. The prevalence and factors associated with antipsychotic polypharmacy in a forensic psychiatric sample. Front Psych. 2020;11:263. doi:10.3389/fpsyt.2020.00263. PMC7247840 32528318

[14] Fleischhacker WW, Uchida H. Critical review of antipsychotic polypharmacy in the treatment of schizophrenia. Int J Neuropsychopharmacol. 2014;17:1083–93. doi:10.1017/S1461145712000399. 22717078

[15] Gallego JA, Bonetti J, Zhang J, Kane JM, Correll CU. Prevalence and correlates of antipsychotic polypharmacy: a systematic review and meta-regression of global and regional trends from the 1970s to 2009. Schizophr Res. 2012;138:18–28. doi:10.1016/j.schres.2012.03.018. 22534420 PMC3382997

[16] Campforts B, Drukker M, Crins J, Van Amelsvoort T, Bak M. Association between antipsychotic medication and clinically relevant weight change: meta-analysis. BJPsych Open. 2023;9:e18. doi:10.1192/bjo.2022.619. 36651070 PMC9885350

[17] Siskind D, Gallagher E, Winckel K, Hollingworth S, Kisely S, Firth J, et al. Does switching antipsychotics ameliorate weight gain in patients with severe mental illness? A systematic review and meta-analysis Schizophr Bull. 2021;47:948–58. doi:10.1093/schbul/sbaa191. 33547471 PMC8266669

[18] Speyer H, Westergaard C, Albert N, Karlsen M, Stürup AE, Nordentoft M, et al. Reversibility of antipsychotic-induced weight gain: a systematic review and meta-analysis. Front Endocrinol. 2021;12:577919. doi:10.3389/fendo.2021.577919. PMC8355990 34393989

[19] Wang H-H, Cai M, Wang H-N, Chen Y-C, Zhang R-G, Wang Y, et al. An assessor-blinded, randomized comparison of efficacy and tolerability of switching from olanzapine to ziprasidone and the combination of both in schizophrenia spectrum disorders. J Psychiatr Res. 2017;85:59–65. doi:10.1016/j.jpsychires.2016.11.002. 27837658

[20] Kim SH, Ivanova O, Abbasi FA, Lamendola CA, Reaven GM, Glick ID. Metabolic impact of switching antipsychotic therapy to aripiprazole after weight gain: a pilot study. J Clin Psychopharmacol. 2007;27:365–8. doi:10.1097/JCP.0b013e3180a9076c. 17632220

[21] Ried LD, Renner BT, Bengtson MA, Wilcox BM, Wilfred WA. Weight change after an atypical antipsychotic switch. Ann Pharmacother. 2003;37:1381–6. doi:10.1345/aph.1C470. 14519048

[22] Lin C-C, Bai Y-M, Wang Y-C, Chen T-T, Lai I-C, Chen J-Y, et al. Improved body weight and metabolic outcomes in overweight or obese psychiatric patients switched to Amisulpride from other atypical antipsychotics. J Clin Psychopharmacol. 2009;29:529–36. doi:10.1097/JCP.0b013e3181bf613e. 19910716

[23] Montes JM, Rodriguez JL, Balbo E, Sopelana P, Martin E, Soto JA, et al. Improvement in antipsychotic-related metabolic disturbances in patients with schizophrenia switched to ziprasidone. Prog Neuro-Psychopharmacol Biol Psychiatry 2007;31:383–8. doi:10.1016/j.pnpbp.2006.10.002. 17129654

[24] Schuster J-P, Raucher-Chéné D, Lemogne C, Rouillon F, Gasquet I, Leguay D, et al. Impact of switching or initiating antipsychotic treatment on body weight during a 6-month follow-up in a cohort of patients with schizophrenia. J Clin Psychopharmacol. 2012;32:672–7. doi:10.1097/JCP.0b013e31826866db. 22926602

[25] Karayal ON, Glue P, Bachinsky M, Stewart M, Chappell P, Kolluri S, et al. Switching from quetiapine to ziprasidone: a sixteen-week, open-label, multicenter study evaluating the effectiveness and safety of ziprasidone in outpatient subjects with schizophrenia or schizoaffective disorder. J Psychiatr Pract. 2011;17:100–9. doi:10.1097/01.pra.0000396061.05269.c8. 21430488

[26] Mitchell AJ, Vancampfort D, De Herdt A, Yu W, De Hert M. Is the prevalence of metabolic syndrome and metabolic abnormalities increased in early schizophrenia? A comparative meta-analysis of first episode, untreated and treated patients. Schizophr Bull. 2013;39:295–305. doi:10.1093/schbul/sbs082. 22927670 PMC3576152

[27] Huhn M, Nikolakopoulou A, Schneider-Thoma J, Krause M, Samara M, Peter N, et al. Comparative efficacy and tolerability of 32 oral antipsychotics for the acute treatment of adults with multi-episode schizophrenia: a systematic review and network meta-analysis. Lancet. 2019;394:939–51. doi:10.1016/S0140-6736(19)31135-3. 31303314 PMC6891890

[28] Sabé M, Pallis K, Solmi M, Crippa A, Sentissi O, Kaiser S. Comparative effects of 11 antipsychotics on weight gain and metabolic function in patients with acute schizophrenia: a dose-response meta-analysis. J Clin Psychiatry. 2023;84. doi:10.4088/JCP.22r14490. 36752753

[29] Ratzoni G, Gothelf D, Brand-Gothelf A, Reidman J, Kikinzon L, Gal G, et al. Weight gain associated with olanzapine and risperidone in adolescent patients: a comparative prospective study. J Am Acad Child Adolesc Psychiatry. 2002;41:337–43. doi:10.1097/00004583-200203000-00014. 11886029

[30] Schimmelmann BG, Mehler-Wex C, Lambert M, Schulze-zur-Wiesch C, Koch E, Flechtner HH, et al. A prospective 12-week study of quetiapine in adolescents with schizophrenia spectrum disorders. J Child Adolesc Psychopharmacol. 2007;17:768–78. doi:10.1089/cap.2007.0048. 18315449

[31] Tek C, Kucukgoncu S, Guloksuz S, Woods SW, Srihari VH, Annamalai A. Antipsychotic-induced weight gain in first-episode psychosis patients: a meta-analysis of differential effects of antipsychotic medications: weight gain in FEP patients. Early Interv Psychiatry. 2016;10:193–202. doi:10.1111/eip.12251. 25962699 PMC5589463

[32] Zipursky RB, Gu H, Green AI, Perkins DO, Tohen MF, McEvoy JP, et al. Course and predictors of weight gain in people with first-episode psychosis treated with olanzapine or haloperidol. Br J Psychiatry. 2005;187:537–43. doi:10.1192/bjp.187.6.537. 16319406

[33] Faries DE, Ascher-Svanum H, Nyhuis AW, Kinon BJ. Switching from risperidone to olanzapine in a one-year, randomized, open-label effectiveness study of schizophrenia. Curr Med Res Opin. 2008;24:1399–405. doi:10.1185/030079908X297385. 18397549

[34] Godleski LS, Goldsmith LJ, Vieweg WV, Zettwoch NC, Stikovac DM, Lewis SJ. Switching from depot antipsychotic drugs to olanzapine in patients with chronic schizophrenia. J Clin Psychiatry. 2003;64:119–22. doi:10.4088/JCP.v64n0203. 12633119

[35] Ascher-Svanum H, Stensland MD, Kinon BJ, Tollefson GD. Weight gain as a prognostic indicator of therapeutic improvement during acute treatment of schizophrenia with placebo or active antipsychotic. J Psychopharmacol (Oxf). 2005;19:110–7. doi:10.1177/0269881105058978. 16280344

[36] Czobor P, Volavka J, Sheitman B, Lindenmayer J-P, Citrome L, McEvoy J, et al. Antipsychotic-induced weight gain and therapeutic response: a differential association: J Clin Psychopharmacol. 2002;22:244–51. doi:10.1097/00004714-200206000-00003. 12006893

[37] Leadbetter R, Shutty M, Pavalonis D, Vieweg V, Higgins P, Downs M. Clozapine-induced weight gain: prevalence and clinical relevance. Am J Psychiatry. 1992;149:68–72. doi:10.1176/ajp.149.1.68. 1728188

[38] Luckhoff H, Phahladira L, Scheffler F, Asmal L, Du Plessis S, Chiliza B, et al. Weight gain and metabolic change as predictors of symptom improvement in first-episode schizophrenia spectrum disorder patients treated over 12 months. Schizophr Res. 2019;206:171–6. doi:10.1016/j.schres.2018.11.031. 30503765

[39] Doporučené postupy psychiatrické péče Psychiatrické společnosti ČLS JEP. https://postupy-pece.psychiatrie.cz/specialni-psychiatrie/f2-schizofrenie/lecba-akutni-epizody-schizofrenie; Published 2018. Accessed January 8, 2024.

[40] National Collaborating Centre for Mental Health (UK). Psychosis and schizophrenia in adults: Treatment and management: Updated edition 2014. London: National Institute for Health and Care Excellence (UK); 2014. 25340235

[41] Remington G, Addington D, Honer W, Ismail Z, Raedler T, Teehan M. Guidelines for the pharmacotherapy of schizophrenia in adults. Can J Psychiatr. 2017;62:604–16. doi:10.1177/0706743717720448. PMC5593252 28703015

[42] American Psychiatric Association, editor. The American Psychiatric Association practice guideline for the treatment of patients with schizophrenia. 3rd ed. Washington, DC: American Psychiatric Association; 2021.

[43] Galletly C, Castle D, Dark F, Humberstone V, Jablensky A, Killackey E, et al. Royal Australian and New Zealand College of Psychiatrists clinical practice guidelines for the management of schizophrenia and related disorders. Aust N Z J Psychiatry. 2016;50:410–72. doi:10.1177/0004867416641195. 27106681

[44] Premier épisode psychotique - Choix de l’antipsychotique. https://www.chuv.ch/fileadmin/sites/dp/documents/dp-uppc-premier-episode-psychotique.pdf; Published 2024. Accessed March 30, 2024.

[45] Ganguly R, Kotzan JA, Miller LS, Kennedy K, Martin BC. Prevalence, trends, and factors associated with antipsychotic polypharmacy among Medicaid-eligible schizophrenia patients, 1998–2000. J Clin Psychiatry. 2004;65:1377–88. doi:10.4088/JCP.v65n1013. 15491242

[46] Faries D, Ascher-Svanum H, Zhu B, Correll C, Kane J. Antipsychotic monotherapy and polypharmacy in the naturalistic treatment of schizophrenia with atypical antipsychotics. BMC Psychiatry. 2005;5:26. doi:10.1186/1471-244X-5-26. 15921508 PMC1156914

[47] Barbui C, Nosè M, Mazzi MA, Thornicroft G, Schene A, Becker T, et al. Persistence with polypharmacy and excessive dosing in patients with schizophrenia treated in four European countries: Int Clin Psychopharmacol. 2006;21:355–62. doi:10.1097/01.yic.0000224785.68040.43. 17012982

[48] Correll CU, Frederickson AM, Kane JM, Manu P. Does antipsychotic polypharmacy increase the risk for metabolic syndrome? Schizophr Res. 2007;89:91–100. doi:10.1016/j.schres.2006.08.017. 17070017 PMC2718048

[49] Tiihonen J, Taipale H, Mehtälä J, Vattulainen P, Correll CU, Tanskanen A. Association of antipsychotic polypharmacy vs monotherapy with psychiatric rehospitalization among adults with schizophrenia. JAMA Psychiatry. 2019;76:499. doi:10.1001/jamapsychiatry.2018.4320. 30785608 PMC6495354

[50] Correll CU, Rummel-Kluge C, Corves C, Kane JM, Leucht S. Antipsychotic combinations vs monotherapy in schizophrenia: a meta-analysis of randomized controlled trials. Schizophr Bull. 2009;35:443–57. doi:10.1093/schbul/sbn018. 18417466 PMC2659301

[51] Taylor M, Cavanagh J, Hodgson R, Tiihonen J. Examining the effectiveness of antipsychotic medication in first-episode psychosis. J Psychopharmacol (Oxf). 2012;26:27–32. doi:10.1177/0269881112439252. 22337711

[52] Crossley NA, Constante M, McGuire P, Power P. Efficacy of atypical *v.* typical antipsychotics in the treatment of early psychosis: meta-analysis. Br J Psychiatry. 2010;196:434–9. doi:10.1192/bjp.bp.109.066217. 20513851 PMC2878818

[53] Zhang J-P, Gallego JA, Robinson DG, Malhotra AK, Kane JM, Correll CU. Efficacy and safety of individual second-generation vs. first-generation antipsychotics in first-episode psychosis: a systematic review and meta-analysis. Int J Neuropsychopharmacol. 2013;16:1205–18. doi:10.1017/S1461145712001277. 23199972 PMC3594563

