# Supplementary material for: Trajectories of daily antipsychotic use and weight gain in people hospitalized for the first episode of psychosis
Source: Eur Psychiatry. 2024 Sep 26;67(1):e59. doi: 10.1192/j.eurpsy.2024.1761 (PMC11457116; doi:10.1192/j.eurpsy.2024.1761)
Supplement: Vochoskova et al. supplementary material [file S0924933824017619sup001.docx]

**Supplementary materials**

| rank | type of antipsychotic medication |
| --- | --- |
| 1 | ziprasidone |
| 2 | haloperidol |
| 3 | aripiprazole |
| 4 | lurasidone |
| 5 | cariprazine |
| 6 | amisulprid |
| 7 | First generation antipsychotics (melperone, zuclopenthixol, flupenthixole, levopromazine) |
| 8 | paliperidone |
| 9 | risperidone |
| 10 | quetiapine |
| 11 | clozapine |
| 12 | olanzapine |
| 0 | no antipsychotics |

**Supplementary table 1** Ranked antipsychotic medications based on the strength of their association with body weight using whole numbers from 1 to 12 according to recent network metanalyses, see references [11,27,28].

**Alternative approach for expresing medication ranking with polypharmacy trajectories using average weight gain associated with each medication:**

Neither medication weight gain slope (t(69)=-0.30, p=0.765) nor intercept (t(69)=0.08, p=0.934) were significantly associated with change in BMI, when controlling for all other predictors (dose slope, dose intercept, baseline BMI, treatment duration, age, sex).
